# Genome-wide Association Study (GWAS) of mesocotyl elongation based on re-sequencing approach in rice

**DOI:** 10.1186/s12870-015-0608-0

**Published:** 2015-09-11

**Authors:** Jinhong Wu, Fangjun Feng, Xingming Lian, Xiaoying Teng, Haibin Wei, Huihui Yu, Weibo Xie, Min Yan, Peiqing Fan, Yang Li, Xiaosong Ma, Hongyan Liu, Sibin Yu, Gongwei Wang, Fasong Zhou, Lijun Luo, Hanwei Mei

**Affiliations:** Shanghai Agrobiological Gene Center; Shanghai Research Station of Crop Gene Resource & Germplasm Enhancement, Chinese Ministry of Agriculture, Shanghai, 201106 China; National Key Laboratory of Crop Genetic Improvement, Huazhong Agricultural University, Wuhan, China; Life Science and Technology Center, China National Seed Group Co., Ltd, Wuhan, China

## Abstract

**Background:**

Mechanized dry seeded rice can save both labour and water resources. Rice seedling establishment is sensitive to sowing depth while mesocotyl elongation facilitates the emergence of deeply sown seeds.

**Results:**

A set of 270 rice accessions, including 170 from the mini-core collection of Chinese rice germplasm (C Collection) and 100 varieties used in a breeding program for drought resistance (D Collection), was screened for mesocotyl lengths of seedlings grown in water (MLw) in darkness and in 5 cm sand culture (MLs). Twenty six accessions (10.53 %) have MLw longer than 1.0 cm. Eleven accessions had the highest mesocotyl lengths, i.e. 1.4 – 5.05 cm of MLw and 3.0 – 6.4 cm in 10 cm sand culture, including 7 upland landraces or varieties. The genotypic data of 1,019,883 SNPs were developed by re-sequencing of those accessions. A whole-genome SNP array (Rice SNP50) was used to genotype 24 accessions as a validation panel, giving 98.41 % of consistent SNPs with the re-sequencing data in average. GWAS based on compressed mixed linear model was conducted using GAPIT. Based on a threshold of -log(P) ≥8.0, 13 loci were associated to MLw on rice chromosome 1, 3, 4, 5, 6 and 9, respectively. Three associated loci, on chromosome 3, 6, and 10, were detected for MLs. A set of 99 associated SNPs for MLw, based on a compromised threshold (−log(P) ≥7.0), located in intergenic regions or different positions of 36 annotated genes, including one cullin and one growth regulating factor gene.

**Conclusions:**

Higher proportion and extension of elongated mesocotyls were observed in the mini-core collection of rice germplasm and upland rice landraces or varieties, possibly causing the correlation between mesocotyl elongation and drought resistance. GWAS found 13 loci for mesocotyl length measured in dark germination that confirmed the previously reported co-location of two QTLs across populations and experiments. Associated SNPs hit 36 annotated genes including function-matching candidates like cullin and GRF. The germplasm with elongated mesocotyl, especially upland landraces or varieties, and the associated SNPs could be useful in further studies and breeding of mechanized dry seeded rice.

**Electronic supplementary material:**

The online version of this article (doi:10.1186/s12870-015-0608-0) contains supplementary material, which is available to authorized users.

## Background

The rice cultivation system based on transplanting of seedlings from nursery to puddled fields, namely transplanting rice (TPR), was popular in China and other Asian countries as the major rice production regions. TPR has several advantages like higher yield potential, convenience in application of fertilizers and pesticides, control of weeds, etc. But TPR requires large amount of water, labour and energy costs in preparing the field, and uprooting and transplanting the seedlings. Changes in the method of rice establishment was expected in response to the rising scarcity of land, water and labour [1, 2]. Seedling-throwing or mechanized transplanting, wet or water direct seeding can save labour costs. However, preparing the puddled fields still requires large amounts of water, together with higher costs from labour, farm animals or machines than the preparation of dry fields. Manual dry seeding can save water, but are labour costing. So mechanized dry seeding is probably the most efficient way of rice seedling establishment, saving 30 % labour than machine-transplanting rice (MTPR) as estimated in Korean trials [3].

In rainfed areas or areas of inadequate irrigation, transplanting rice could completely fail or delay in years with less and/or delayed rainfall. As an example, a minimum of 600 mm of cumulative rainfall was required to complete field puddling and transplanting of rice in the Philippines, much higher than 150 mm cumulative rainfall required by dry seeding [4]. In 1 year of every 4 years, a delay of 20 days for dry seeding could happen, much shorter than 40-day delay for transplanting [5]. MDSR has been widely adopted and will expand to much larger area if effective managements are available to control weeds and to maintain uniform plant density, e.g. fine tillage, better land levelling, more appropriate seed placement, improved nutrient application, varieties with higher seedling vigor and lodge resistance [6]. So far, the appropriate techniques are not fully available yet to ensure the perfect seedling establishments.

Rapid and well seedling establishment is important for weed competitiveness and good harvesting of DSR, determined by sowing depth and a few other factors. The seedling establishment and shoot dry weight were critically affected by the depths of soil and water layer in lowland wet seeded rice [7]. Hanviriyapant *et al*. reported the well establishment and strong seedlings of a tall, vigorous-growing cultivar and higher sensitivity of semidwarf cultivar to sowing depth and time of sowing after irrigation [8]. An experiment of gradient sowing depths showed that the seedling establishment of wheat was not affected by sowing depths from 2.3 to 8.3 cm, but declined to about 6 % at 14.3 cm [9].

Elongation of both mesocotyl and coleoptile can facilitate the seedling establishment of rice when sown deep in soil or under water layer [10, 11]. Mgonja *et al*. reported the association between mesocotyl elongation and seedling vigor [12]. Alibu *et al*. found that coleoptile length was more enhanced under submergence while mesocotyl elongated more in soil-sand culture. Sown 8 cm deep, the emergence of only a few genotypes was determined by varied mesocotyl elongation, not the variation of coleoptile lengths [13], similar to an early observation in *indica* rice [14]. Mesocotyl elongation has been found to be the cause of deep-seeding tolerance in maize [15, 16].

Mesocotyl elongation has been measured in several sets of germplasm, e. g. 128 weedy rice or Korean cultivars [11], 27 diverse cultivars [17], near 100 rice accessions [18] and 1500 accessions [19]. Low percentage of rice germplasm has highly elongated mesocotyl (e. g. longer than 1.0 cm). Genetic analysis showed that mesocotyl length had high heritability [17], but was controlled by different genetic effects [20, 21]. Linkage mapping found 3–8 QTLs for mesocotyl length of rice seedlings in different populations [22–27]. Two QTLs on rice chromosome 1 and 3 were repeatedly detected and showed large effects across different experiments.

Genome-wide association study (GWAS) based on SSR [28] or single nucleotide polymorphism (SNP) markers [29–33] has been widely used in model plant species including rice. Extremely high resolution can be achieved by dense SNPs identified in diverse germplasm panels based on the 2nd generation genome sequencing or SNP array approaches [29–35]. In this study, GWAS based on re-sequencing approach was conducted in a set of rice landraces or varieties for mesocotyl elongation as a key character enhancing rice seedling emergence, especially after dry seeding with relatively higher sowing depth.

## Results

### Phenotypic variations of mesocotyl elongation among rice germplasm accessions

A wide range of mesocotyl lengths in different rice germplasm accessions, from almost no elongation to a maximum of 5.05 cm, were observed in the dark germination experiment. Mesocotyl length varied from nearly zero to a maximum of 2.05 cm among those rice accessions when measured in 5 cm sand culture. ANOVA showed highly significant variance among rice germplasm accessions, together with less or no significant variance between replications for ML in dark germination with water (MLw) and ML in sand culture (MLs) (Table 1).

**Table 1:** ANOVA of mesocotyl length of rice seedlings in dark germination in water (MLw) or 5 cm sand culture (MLs)

Traits	Sources	Df	SS	MS	F value	*P* value
MLw (cm)	Line	246	207.2965	0.8427	104.99	0.0000
	Rep	1	0.0452	0.0452	5.63	0.0184
	Residuals	246	1.9744	0.0080		
MLs (cm)	Line	246	75.3382	0.3050	6.17	0.0000
	Rep	1	0.0046	0.0046	0.09	0.7610
	Residuals	246	1216.7900	4.9500		

**Fig. 1:**
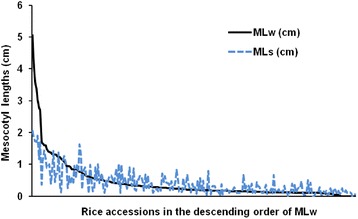
Varied mesocotyl lengths among rice landraces or varieties, measured in seedlings from dark germination in water (MLw) or 5 cm sand culture (MLs)

As shown in Fig. 1, only a low proportion of germplasm accessions had largely elongated mesocotyl. The MLw of 26, 29 and 192 accessions were higher than 1.0 cm, in the range of 0.5–1.0 cm and shorter than 0.5 cm, respectively. MLs showed similar general trend with MLw, but had some deviation around MLw (Fig. 1). The mesocotyl lengths measured in dark germination (MLw) and in sand culture (MLs) had highly significant correlation (*r* = 0.784**; Additional file 1: Table S1).

**Fig. 2:**
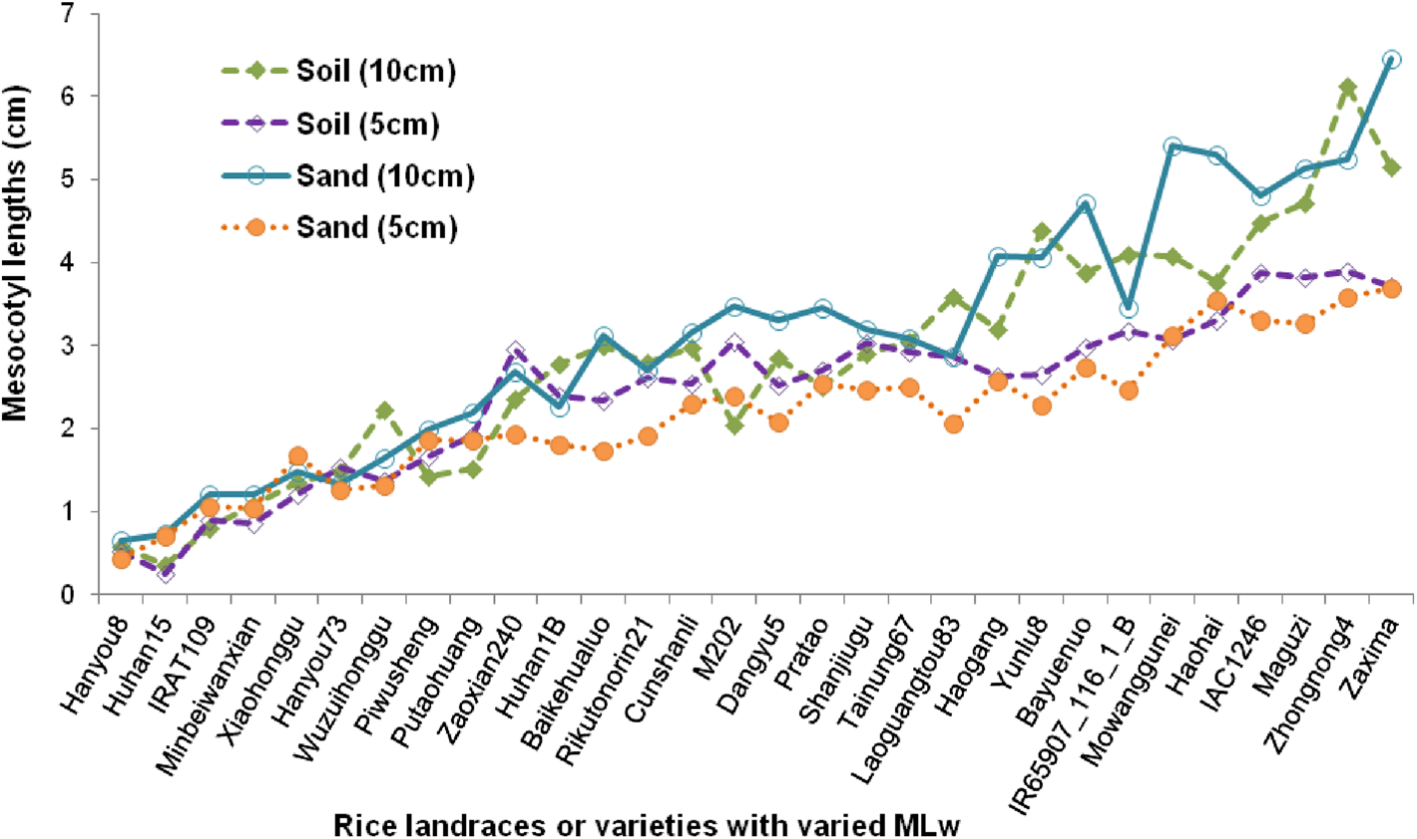
Mesocotyl lengths of 30 rice germplasm accessions measured in sand or soil culture with two seeding depths

A third experiment was conducted to confirm previous results and to check the reaction of mesocotyl elongation to higher depth of sand or soil covering layers, using 30 landraces or varieties representing accessions with low, medium and high mesocotyl elongation. As sorted by MLw on the axis of abscissa (Fig. 2), ascending lines showed consistent trends between the measurements of mesocotyl lengths in all experiments. The seedlings had similar mesocotyl lengths in either sand or soil culture. The reaction of mesocotyl elongation to two seeding depths showed different patterns among rice accessions. The first 10 accessions (on the left in the chart) had almost same mesocotyl lengths for both depths, i.e. no more increase under 10 cm sand culture as a more favoured condition, implying that the measurements here represented the maximum capacity of mesocotyl elongation of those accessions. Another 10 accessions (in the middle) had a little longer mesocotyl lengths under 10 cm than under 5 cm covering layers, suggesting their maximum capacity up to 2.5–3 cm that was equivalent to or a little higher than the detectable limit in experiment of 5 cm sand or soil culture. For the last 10 accessions, mesocotyl lengths were higher in 10 cm than in 5 cm depth. It is obvious that those landraces or varieties had capacities of mesocotyl elongation from 3 to 6 cm, fully expressed in 10 cm, but not in 5 cm culture. The low measurements (2–3 cm) in 5 cm sand or soil culture were perhaps the result of light inhibition after the emergence of coleoptiles or leaves of the seedlings.

Eleven rice accessions, TAINUNG 67, HAOGANG, YUNLU 8, BAYUENUO, IR65907-116-1-B, MOWANGGUNEI, HAOHAI, IAC1246, MAGUZI, ZHONGNONG 4 and ZAXIMA, possessed high mesocotyl lengths in all experiments, i. e. 1.4 – 5.05 cm in dark germination and 3.0 – 6.4 cm in 10 cm soil or sand culture. Among them, seven accessions were upland landraces (HAOGANG, MOWANGGUNEI, HAOHAI and ZAXIMA) or upland varieties (YUNLU 8, IR65907-116-1-B and IAC1246).

### SNP validation and population structure analysis

A subset of 24 accessions, including 9 from C collection and 15 from D collection, were genotyped using the RiceSNP50 whole-genome SNP array [31]. There are 10,851 SNP loci shared by the genotypic data sets from re-sequencing SNP calling and SNP array. Each accession has effective data on 8,313–10,746 common SNP loci after excluding loci with missing data in either SNP calling or array. The accuracy of SNP calling and missing genotype imputation, represented by the percentage of consistent SNPs in total number of common loci, reached 98.41 % in average and ranged from 97.01 to 99.53 % for each accession (Additional file 2: Table S2).

**Fig. 3:**
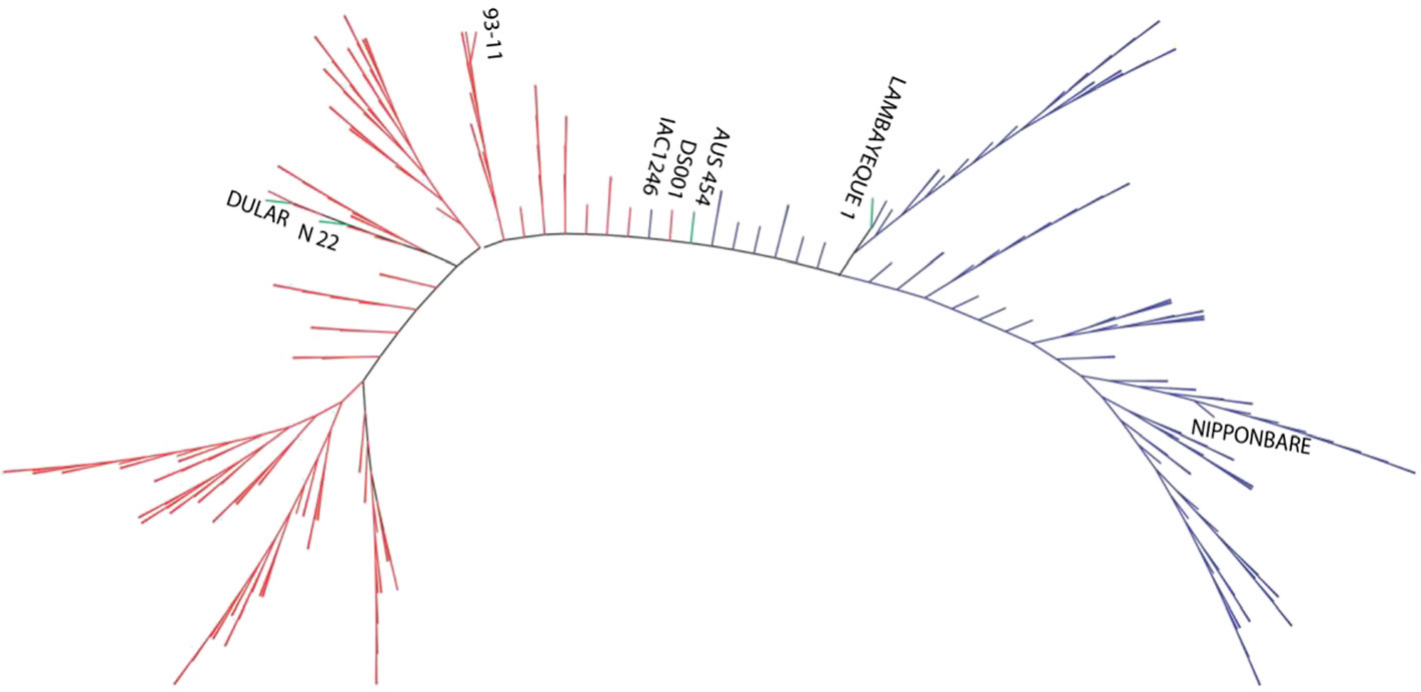
Neighbor joining tree of 270 rice accessions showed a two-subpopulation structure in consistence with the classification of *indica* (in *red*) and *japonica* (in *blue*) subspecies. Four *aus* accessions (in *green*) were grouped into two subpopulations

The population structure was estimated using a subset of 144,995 SNP loci with less than 10 % missing data in D collection before imputation (as the total SNP number called from the sequencing reads of the accessions in the D collection is much lower than that in the C collection). Using genotypic data before imputation could avoid the possible influence from imputed values on genetic distance and LD levels. A two sub-population structure, highly matching the two subspecies in rice, was observed among those accessions in this study (Fig. 3; Additional file 3: Figure S1). Among 4 *aus* accessions, DULAR and N22 were grouped into *indica* while AUS 454 and LAMBAYEQUE into *japonica* subpopulation.

### Genome-wide association study (GWAS)

**Fig. 4:**
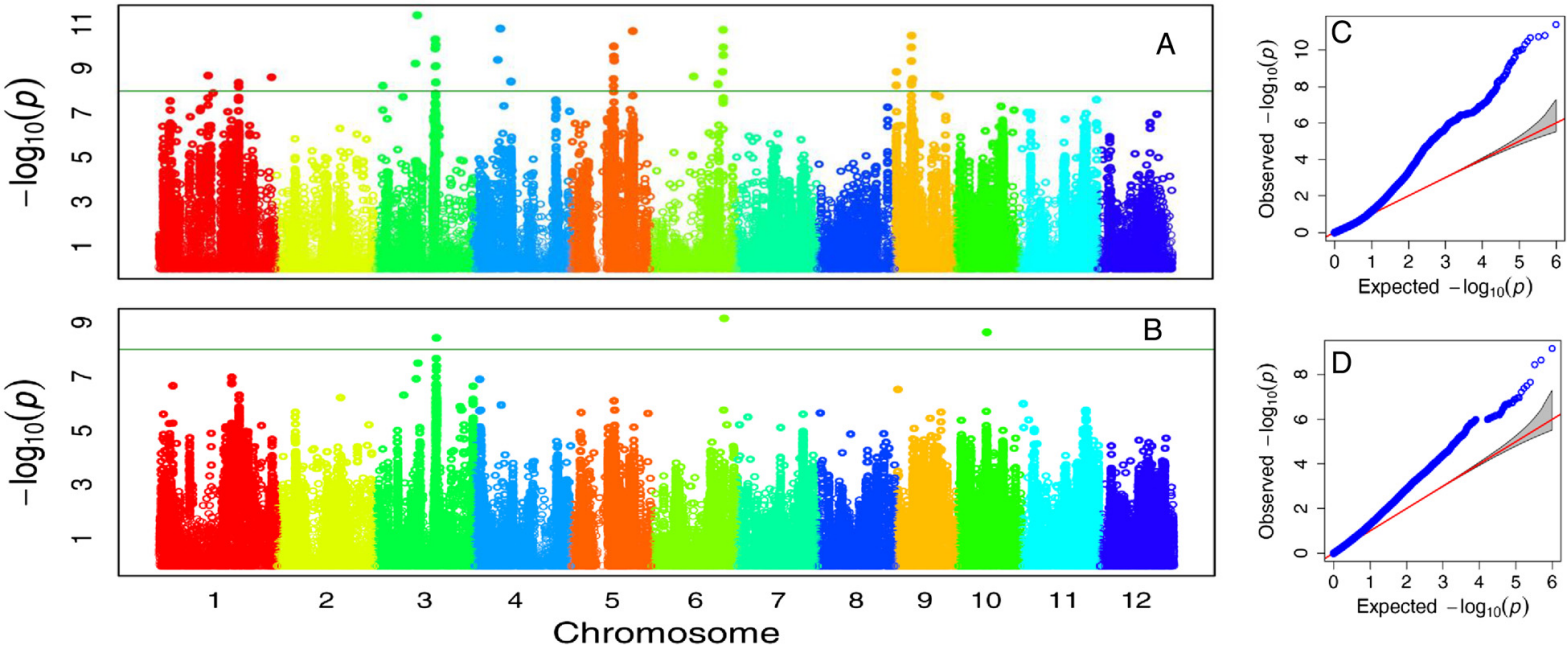
Manhattan plots of genome-wide association mapping for mesocotyl lengths measured in dark germination with water (MLw, **a**) and in 5 cm sand culture (MLs, **b**) and Quantile-Quantile plots for MLs (**c**) and MLs (**d**)

Forward model selection procedure provided the largest Bayesian information criteria (BICs) for both traits when zero PCs/covariates were included in the GWAS models (Additional file 4: Table S3). This result suggested that the PCs estimated from SNP data had weak covariance with the phenotypic data. Using -log(P) ≥8.0 as the threshold at a significant level of 0.01 after Bonferroni multiple test correction, a total of 13 loci were declared to have highly significant association with the mesocotyl lengths (MLw). Those associated loci were located on 6 chromosomes of rice, including 3, 3, 1, 2, 2, 2 loci on chromosome 1, 3, 4, 5, 6 and 9, respectively (Fig. 4a). Seven peaks with -log(P) values larger than 10 in Manhattan plot indicated very strong signals of association between the trait and the chromosomal regions, especially four regions on chromosome 3, 5, 6 and 9 which host sharp -log(P) peaks.

The Manhattan plot of MLs shows totally different pattern (Fig. 4b). Only three associated SNPs were detected at the significant level of -log(P) ≥8.0, including two SNPs locating in the same regions associated to MLw on chromosome 3 and 6, one SNP on chromosome 10 with no association to MLw.

As Bonferroni correction was recognized to be too conservative [36], a compromised threshold of –log(P) ≥7.0 was used to screen out a set of 99 SNPs associating to MLw and 7 SNPs to MLs (Additional file 5: Table S4). Among MLw associated SNPs, 52, 16, 24, 3, 3, 1 SNPs located in intergenic regions, intron, promoter, CDS-synonymous, CDS-nonsynonymous and 5′ UTR regions of 36 annotated genes, respectively. Two MLs associated SNPs hit the promoter region of LOC_Os03g40390 while another SNP and the remaining four SNPs located in the intron of LOC_Os10g20860 and the intergenic regions, respectively.

In about 15.7Kb interval (29288539-29304267) on rice chromosome 1, five MLw associated SNPs located in the promoter, CDS-nonsynonymous or intergenic regions of three putative genes (LOC_Os01g50970, LOC_Os01g50980, LOC_Os01g50990). Those genes have been annotated as expressed protein with unknown function, putatively expressed cullin and FBD domain containing protein, respectively. One associated SNP (0430137498) located in the promoter of rice gene LOC_Os04g51190, annotated as a growth-regulating factor.

## Discussion

### Retrieving the character of mesocotyl elongation to develop varieties for mechanized dry seeded rice

In the past several decades, many labour-saving methods of seedling establishment have been developed and widely used in rice production in Asian countries where hand transplanting rice became common during 1950–70s. Among them, mechanized dry seeded rice (MDSR) is probably the system using the least water and labour resource [3–5]. As the majority of modern rice varieties were developed for transplanting system in irrigated environments, their performance has not been optimized for direct seeding, especially in drought-prone environments. Early maturing, high-yielding rice varieties that can withstand drought and compete with weeds are urgently required in the dry-seeded rice system. In this case, well establishment and vigorous growth of the rice seedlings become very important [4].

In order to obtain quick and uniform seedling emergence, shallow sowing with a narrow range of depth (e.g. 2–3 cm) is required in drill seeding for most semidwarf rice varieties. Seedling establishment decreases remarkably, together with the delayed seedling emergence and poor early growth, when seeding depth is higher than 5 cm [3]. But shallowly sown seeds are vulnerable to bird damage while the derived plants are possibly sensitive to lodging at late stage [36]. In drought prone areas, the quick lost of moisture in shallow soil layer would cause delayed or failed seed germination and seedling emergence. This is the major reason why the period from pre-irrigation to sowing has critical influence on seedling establishment of DSR [8]. Narrow tolerant range of seeding depth will cause high risk of inadequate management in mechanized seeding if the soil was not finely tilled and levelled or the seed drill did not give precise seed placement. So rice varieties with tolerance to varied seeding depth, would reduce such kind of risk or additional requirements to farm machinery, then facilitate the expanding of mechanized dry seeded rice.

An early observation confirmed the association of mesocotyl elongation with seedling vigor in rice [12] and a wide range of genetic variation of this trait among rice germplasm [11, 13, 17–19], even though the percentage of germplasm with mesocotyl length higher than 1.0 cm was low, e.g. less than 1 % in a set of 1500 accessions [19]. In this study, 26 accessions had mesocotyl length (MLw) higher than 1.0 cm, showing much higher percentage (10.53 %) than previous reports (Fig. 1). Among 11 accessions with most elongated mesocotyl in this study, there are 7 upland accessions (4 landraces and 3 varieties), accounting for a quite high proportion. Larger genetic variation could be expected in core or mini-core collection of germplasm. And it seems reasonable that more upland rice accessions have highly elongated mesocotyl [18].

A few publications described the failed emergence of semidwarf rice varieties and/or the successful emergence of tall, vigorously growing varieties when sown deep [8, 10]. It should be true that most modern rice varieties, developed for transplanting cultivation, have lost the character of mesocotyl elongation. But an important question is whether mesocotyl elongation is tightly linked to plant height. Mgonja *et al*. found no correlation between mesocotyl elongation and characters of mature plants like plant height and internode length L1 [20]. In this study, the same set of rice accessions were evaluated in field for drought resistance using water regimes (data not shown). Both MLw and MLs are correlated to plant height in both conditions (*r* = 0.250 ~ 0.349; *P* ≤ 0.01; Additional file 1: Table S1); correlated to grain yield and spikelet fertility in drought treatment, but not in well watered condition. These results did not necessarily indicate the linkage or pleiotropism of loci controlling mesocotyl elongation and plant height or drought resistance. It is more likely the consequences of the high proportion of upland landraces or varieties in the population which had longer mesocotyl, higher plant height and drought resistance at the same time. So development of semidwarf varieties possessing both mesocotyl elongation and drought resistance is necessary for mechanized dry seeded rice and achievable by using those potential germplasm screened in this study.

### Mesocotyl elongation QTLs and candidate genes 

Among 3–8 QTLs for mesocotyl length reported in different mapping populations [22–27], two QTLs (*qMel-1*, *qMel-3*) on rice chromosome 1 and 3 were repeatedly detectable and showed large effects across experiments [22–24, 26, 27, 37]. Substitution mapping confined *qMel-1* into a 3,799Kb interval from RM5448 to RM5310 and *qMel-3* into a 6,964Kb region from RM3513 to RM1238, containing 490 and 700 putative genes, respectively [27]. In this study, one SNP marker at the bottom of chromosome 1 was associated with MLw (*P* = 2.57E-09), about 0.17 Mb away from the interval of RM5448-RM5310. Strong association signals were detected in *qMel-3* region represented by the sharp -log(P) peaks in the Manhattan plots for both MLw and MLs (Fig. 4), including 3 SNPs within a 50 Kb region. The positions of those associated SNPs were not within, but about 2.59 Mb beyond the interval between RM3513 and RM1238. If confirmed in further studies like candidate gene cloning, the results demonstrate the high power of GWAS based on high dense SNPs.

The threshold of genome-wide association test using a large number of SNP markers remains an issue under controversy. Nakagawa suggested that both standard and adjusted Bonferroni procedures should be abandoned because of reduced statistical power [38]. Controlling of false discovery rate (FDR) was introduced by Benjamini [39] and recommended as a better statistical reference to set the threshold of associated loci. In this study, both *P* values and FDR adjusted P values showed similar effect in locating loci if referring to the peaks of significance above –log(P) ≥6 or –log(FDR adjusted P) ≥3 (Additional file 6: Figure S2A). In general, −log(FDR adjusted P) values increased as –log(P) values did (Additional file 6: Figure S2B). However, −log(FDR adjusted P) values remained unchanged around 3 while –log(P) varied from 6 to 7. Declared at the threshold of –log(FDR adjusted P) ≥3, the number of associated SNPs, 401 for MLw, seems too large. So a compromised threshold at –log(P) ≥7 were used to select significant SNPs (99 for MLw; 7 for MLs). Forty seven SNPs located in different positions of 36 annotated genes (itional file 5, Table S4). Among them, one cullin gene and *OsGRF3* had putative functions related to growth regulation. Cullin proteins was found as part of the scaffolds of multiple E_3_ ligase [40], including the E_3_ ubiquitin ligase SCF^TIR1^ that mediates ubiquitination of auxin/IAA proteins [41]. The first growth regulating factor gene (*OsGRF1*) was identified as a transcript factor in rice, responding to gibberellin (GA) and showing potential regulatory role in stem growth [42]. Choi *et al*. [43] analyzed the expression patterns of *OsGRF1* and its 11 homologs in the rice genome. Seven genes showed induced expression by GA_3_. Almost all *OsGRF* genes had high expression in primary leaves and the highest node containing shoot apical meristem or intercalary meristem and part of the elongation zone. As a candidate gene hit by the associated SNP in our study, *OsGRF3* was the only GRF gene that had strong level of expression in mesocotyls and coleoptiles.

## Conclusions

Higher proportion and extension of mesocotyl elongation were observed in a population of landraces and varieties from the mini-core collection of Chinese rice germplasm and a collection of parental varieties for drought tolerant rice breeding. High proportion of upland rice accessions within those having top mesocotyl lengths (7 of 11 accessions) could be the cause of the correlation between mesocotyl elongation and drought resistance, implying the important role and reservation of this character in upland rice germplasm. GWAS found 13 loci for mesocotyl length measured in dark germination that confirmed the previously reported co-location of two QTLs across populations and experiments. Associated SNPs hit 36 annotated genes including putatively function-matching candidates like cullin and GRF. The germplasm with elongated mesocotyl, especially upland landraces or varieties, and the associated SNPs could be useful in further studies and breeding of mechanized dry seeded rice.

## Methods

### Rice germplasm and phenotypic experiments

The materials used in this study consisted of two sets of rice germplasm. One is part of the mini-core collection of Chinese rice germplasm, provided by Huazhong Agricultural University and China Agricultural University (170 accessions, denoted as C Collection) [33, 44] and a set of varieties collected for the breeding program of water-saving and drought -resistant rice (WDR) [45] by Shanghai Agrobiological Gene Center (100 accessions, denoted as D Collection) (Additional file 7: Table S5).

Two experiments were conducted to measure the mesocotyl length of rice seedlings grown in water (MLw, cm) in darkness or under 5 cm sand layer (MLs, cm) for 10 days. In each of two replications of the dark germination experiment, 20 seeds of each accession were sterilized with 3 % H_2_O_2_ solution, rinsed by tap water three times, submerged in water for pre-soaking by 24 h. Then seeds were put on one layer of filter paper above a sponge sheet in a plastic box with cover (L × W × H = 12 × 12 × 2 cm). The boxes were kept in darkness in carton boxes that were placed in the incubator with constant temperature of 25 °C. The mesocotyl lengths of five normal seedlings from each box were measured using rulers.

The sand culture experiments had two replications that were arranged with 3d interval to allow quick finish of the measurements in each replication. Stainless steel boxes without bottom (L × W × H = 90 × 30 × 30 cm) were placed on a levelled sand bed. After adding 5 cm sand layer, 12 seeds from each accession were placed on sand surface in a single row (about 2 cm apart between seeds) along the width of the box. The space between two rows is about 5 cm. Another 5 cm sand layer was added over the seeds and saturated with water by sprinkler until leaking from the bottom of the boxes. Mesocotyl lengths of 10 seedlings were measured using rulers after all seedlings were taken out from the sand and washed by water. This experiment was conducted in late May to early June in a green house. The air temperature was within the range from 20 to 38 °C while the temperature in sand layer ranged from 20 to 31 °C. There were 247 accessions that had effective phenotypic data of both MLw and MLs after removing accessions with missing data caused by inadequate seed samples or failed germination in one experiment or both experiments.

Thirty accessions, including those with longest MLw and a few accessions with low or moderate mesocotyl elongation, were used in an additional experiment to check the mesocotyl elongation when seeds germinated under 5–10 cm layers of sand or soil. This experiment was conducted using the same boxes and procedure as described above, but setting two depth of cover layer and using dry soil as another medium.

ANOVA and Pearson’s correlation analysis with two-tailed significance were conducted using SPSS v16.0.

### Genotyping by re-sequencing and SNP validation

Whole genome re-sequencing was conducted for two germplasm sets using Solexa Hiseq 2000 system. Accessions in the C Collection and D collection were re-sequenced for 2.5 and 5× average genome coverage, respectively. The same pipelines with similar parameters [33], using the softwares BWA, SAMtools and BCFtools [46, 47], were used to call SNPs from sequencing reads for both collections using the rice reference genome of Nipponbare (MSU Rice Genome Annotation Project Release 6.1) [48, 49]. A merged genotypic data set was built by obtaining the intersectional loci of the two SNP data sets from C and D collections. Imputation procedure was conducted by using FillGenotype program (Filling missing genotype (Fimg), http://www.ncgr.ac.cn/fimg/intr.html) based on K-nearest neighbor (KNN) algorithm, using the default parameters (w = 80, *p* = −7, k = 5, and f = 0.7) [29]. For the whole set of germplasm, the final genotypic data consists of 1,019,883 SNP loci.

In order to evaluate accuracy of SNP calling and imputation pipeline, a high-density whole-genome SNP array, RiceSNP50 [34], was used to genotype a validation panel of 24 accessions including 9 from C collection and 15 from D collection. DNA amplification, fragmentation, chip hybridization, single base extension, staining and scanning were conducted by Life Science and Technology Center, China National Seed Group Co., LTD (Wuhan, China), according to Infinium HD Assay Ultra Protocol (http://www.illumina.com/). The RiceSNP50 array contains about 51K evenly distributed SNP markers [34]. About 43K SNPs with high quality were used in the comparison with the SNP calls from re-sequencing. The percentages of consistent SNP loci were calculated by dividing the number of identical SNPs by the effective SNP number within the common set of SNP loci (*n* = 10,851) between array and SNP calls from re-sequencing (Additional file 2: Table S2).

### Population structure analysis and genome-wide association mapping

Based on a subset of 144,994 SNPs that had less than 10 % missing data in D Collection (with much lower total SNP number than in C collection) before imputation, we used the Dnadist program to generate a pairwise distance matrix that was used to construct the unrooted and unweighted neighbour-joining tree by the Neighbor program from the software PHYLIP (V3.695, http://evolution.genetics.washington.edu/phylip.html) [50]. The exported phylogenetic tree in Newick format was modified in format using an online tool Interactive Tree of Life [51]. In addition, the genetic structure of rice population was estimated by the model-based program STRUCTURE version 2.3.4 (http://pritch.bsd.uchicago.edu/structure.html) [52, 53]. Adopting an admixture model allowing for correlated allele frequencies among populations, with no linkage model, we used the run-length parameters as the burn-in period of 2,000 and the number of MCMC replications after burn-in of 5,000. Ten independent simulations using K-value ranging from 2 to 11, with eight replications, yielded consistent results. The inferred groups between successive K values were decided to identify the real number of clusters of individuals based on Evanno’s methods [54].

As the majority of the germplasm accessions in this study are landraces or varieties from China (Additional file 7: Table S5), most accessions could be classified into *indica* or *japonica* subspecies, according to their registration information from the databases like China National Rice Data Center (http://www.ricedata.cn/variety/) and the International Rice Information System (http://www.iris.irri.org/germplasm2/), together with the clustering results of this study. Only four accessions were specified as *aus* type. Population structure estimation, i.e. calculation of PCA and Kinship (K) matrixes, and genome-wide association analysis (GWA) based on the compressed mixed linear model [55] were conducted using the R package of Genomic Association and Prediction Integrated Tool (GAPIT) [56]. A forward model selection procedure was run to determine if any and how many PCs/covariates should be included in association mapping.

The whole set of 1,019,883 SNPs were used in association mapping, setting a minor allele frequency (MAF) criterion of 5 %. A genome-wide threshold of -log(P) = 8.0, calculated from the formula of “-log10(0.01/effective number of SNPs)”, i.e. the threshold at a significant level of 1 % after Bonferroni multiple test correction (0.01/1019883). As the Bonferroni correction probably had low power, false discovery rate (FDR) [39] was recommended as a better method to set the significant level [38]. The effects of screening significant SNPs associated to MLw based on both -log(P) and -log(FDR adjusted P) were compared (Additional file 6: Figure S2). A compromised threshold at -log(P) ≥7.0 was used to screening SNPs in candidate gene annotation.

## Availability of supporting data

The raw Illumina sequencing data from this study have been submitted to NCBI Sequence Read Archive (SRA) under the accession number PRJNA171289 [30] and PRJNA260762.

## Additional files

Additional file 1: Table S1. Pearson correlation coefficients between mesocotyl elongation and agronomic traits of mature plants measured in phenotyping trial with water regimes. (DOCX 16 kb) Additional file 2: Table S2. Accuracy of SNP calling and missing genotype imputation validated by RiceSNP50 whole-genome SNP array. (DOCX 16 kb) Additional file 3: Figure S1. Two subpopolations defined by STRUCTURE. (DOCX 51 kb) Additional file 4: Table S3. Model selection results for GWAS of rice mesocotyl lengths in two experiments. (DOCX 15 kb) Additional file 5: Table S4. Annotation of candidate genes anchored by associated SNPs. (XLSX 34 kb) Additional file 6: Figure S2. Distribution of –log(P) and –log(FDR adjusted P) values of SNPs with –log(FDR adjusted P) ≥3.0 (A) and the parallel changes of both parameters estimated in GWAS of MLw. (DOCX 126 kb) Additional file 7: Table S5. List of rice landraces or varieties used in this study. (DOCX 33 kb)

## Abbreviations

DSR: Direct seeded rice
GWAS: Genome-wide association study
MAF: Minor allele frequency
MDSR: Mechanized dry seeded rice
MLw: Mesocotyl length of rice seedling grown in water in darkness
MLs: Mesocotyl length of rice seedlings grown in 5 cm sand culture
MTPR: Mechanized transplanting rice
PCA: Principle component analysis
QTL: Quantitative trait locus
SNP: Single nuleotide polymorphism
SSR: Microsatellites
TPR: Transplanting rice

## References

[1] Pandey S, Velasco L, Pandey S, Mortimer M, Wade L, Tuong TP, Lopez K, Hardy B (2002). Economics of direct seeding in Asia: patterns of adoption and research priorities. Direct seeding: research issues and opportunities.

[2] Farooq M, Siddique KHM, Rehman H, Aziz T, Lee DJ, Wahid A (2011). Rice direct seeding: experiences, challenges and opportunities. Soil Till Res.

[3] Lee HM, Kim JK, Kim SS, Park ST, Pandey S, Mortimer M, Wade L, Tuong TP, Lopez K, Hardy B (2002). Status of dry-seeding technologies for rice in Korea. Direct seeding: research issues and opportunities.

[4] Mazid MA, Bhuiyan SI, Mannan MA, Wade LJ, Pandey S, Mortimer M, Wade L, Tuong TP, Lopez K, Hardy B (2002). Dry-seeded rice for enhancing productivity of rainfed drought-prone lands: lessons from Bangladesh and the Philippines. Direct seeding: research issues and opportunities.

[5] Saleh AFM, Bhuiyan SI (1995). Crop and rain water management strategies for increasing productivity of rainfed lowland rice systems. Agric Syst.

[6] Pandey S, Velasco L, Suphanchaimat N, Pandey S, Mortimer M, Wade L, Tuong TP, Lopez K, Hardy B (2002). Economics of direct seeding in Northeast Thailand. Direct seeding: research issues and opportunities.

[7] Yamauchi M, Chuong PV (1995). Rice seedling establishment as affected by cultivar, seed coating with calcium peroxide, sowing depth, and water level. Field Crop Res.

[8] Hanviriyapant P, Sherrard JH, Pearson CJ (1987). Establishment of rice determined by interaction between cultivar, sowing depth and time between irrigation and sowing in North West Australia. Field Crop Res.

[9] Kirby EJM (1993). Effect of sowing depth on seedling emergence, growth and development in barley and wheat. Field Crop Res.

[10] Turner FT, Chen CC, Bollich CN (1982). Coleoptile and mesocotyl lengths in semidwarf rice seedlings. Crop Sci.

[11] Chung NJ (2010). Elongation habit of mesocotyls and coleoptiles in weedy rice with high emergence ability in direct-seeding on dry paddy fields. Crop Pasture Sci.

[12] Mgonja MA, Dilday RH, Skinner SL, Collins FC (1988). Association of mesocotyl elongation with seedling vigor in rice. J Ark Acad Sci.

[13] Alibu S, Saito Y, Shiwachi H, Irie K (2011). Relationship between coleoptile and mesocotyl elongation of upland rice (*Oryza sativa* L.) seedlings under submergence and soil-sand culture. Afr J Agric Res.

[14] Takahashi N (1978). Adaptive importance of mesocotyl and coleoptile growth in rice under different moisture regimes. Aust J Plant Physiol.

[15] Troyer AF (1997). The location of genes governing long first internode of corn. Genetics.

[16] Zhang HW, Ma P, Zhao ZN, Zhao GW, Tian BH, Wang JH, Wang GY (2012). Mapping QTL controlling maize deep-seeding tolerance-related traits and confirmation of a major QTL for mesocotyl length. Theor Appl Genet.

[17] Redoña ED, Mackill DJ (1996). Genetic variation for seedling vigor traits in rice. Crop Sci.

[18] Wu MG, Zhang GH, Lin JR, Cheng SH (2005). Screening for rice germplasm with specially-elongated mesocotyl. Rice Sci.

[19] Luo J, Tang SQ, Hu PS, Louis A, Jiao GA, Tang J (2007). Analysis on factors affecting seedling establishment in rice. Rice Sci.

[20] Mgonja MA, Ladeinde TAO, Aken’Ova ME (1994). Genetic analysis of mesocotyl length and its relationship with other agronomic characters in rice (*Oryza sativa* L.). Euphytica.

[21] Lin JR, Zhang GH, Wu MG, Cao LY, Cheng SH (2006). Genetic analysis of mesocotyl elongation in rice (*Oryza sativa* L. subsp. *japonica*). Acta Agron Sin.

[22] Redoña ED, Mackill DJ (1996). Mapping quantitative trait loci for seedling vigor in rice using RFLPs. Theor Appl Genet.

[23] Katsuta-Seki M, Ebana K, Okuno K (1996). QTL analysis for mesocotyl elongation in rice. Rice Genetics Newsletter.

[24] Cao LY, Zhu J, Yan QC, He LB, Wei XH, Cheng SH (2002). Mapping QTLs with epistasis for mesocotyl length in a DH population from *indica*-*japonica* cross of rice (*Oryza sativa*). Chin J Rice Sci.

[25] Huang C, Jiang SK, Feng LL, Xu ZJ, Chen WF (2010). QTL analysis for mesocotyl length in rice (*Oryza sativa* L.). Acta Agron Sin.

[26] Lee HS, Kang JW, Chung NJ, Choi KS, Ahn SN (2012). Identification of molecular markers for mesocotyl elongation in weedy rice. Korean J Breed Sci.

[27] Lee HS, Sasaki K, Higashitani A, Ahn SN, Sato T (2012). Mapping and characterization of quantitative trait loci for mesocotyl elongation in rice (*Oryza sativa* L.). Rice.

[28] Li XB, Yan WG, Agrama H, Jia LM, Jackson A, Moldenhauer K, Yeater K, McClung K, Wu DX (2012). Unraveling the complex trait of harvest index with association mapping in rice (Oryza sativa L.). PLoS ONE.

[29] Huang XH, Wei XH, Sang T, Zhao Q, Feng Q, Zhao Y, Li CY, Zhu CR, Lu TT, Zhang ZW, Li M, Fan DL, Guo YL, Wang AH, Wang L, Deng LW, Li WJ, Lu YQ, Weng QJ, Liu KY, Wang T, Zhou TY, Jing YF, Li W, Lin Z, Buckler ED, Qian Q, Zhang QF, Li JY, Han B (2010). Genome-wide association studies of 14 agronomic traits in rice landraces. Nat Genet.

[30] Huang XH, Zhao Y, Wei XH, Li CY, Wang AH, Zhao Q, Li WJ, Guo YL, Deng LW, Zhu CR, Fan DL, Lu YQ, Weng QJ, Liu KY, Zhou TY, Jing YF, Si LZ, Dong GJ, Huang T, Lu TT, Feng Q, Qian Q, Li JY, Han B (2012). Genome-wide association study of flowering time and grain yield traits in a worldwide collection of rice germplasm. Nat Genet.

[31] Huang XH, Kurata N, Wei XH, Wang ZX, Wang AH, Zhao Q, Zhao Y, Liu KY, Lu HY, Li WJ, Guo YL, Lu YQ, Zhou CC, Tan DL, Weng QJ, Zhu CR, Huang T, Zhang L, Wang YC, Feng L, Furuumi H, Kubo T, Miyanbayashi T, Yuan XP, Xu Q, Dong GJ, Zhan QL, Li CY, Fujiyama A, Toyoda A, Lu TT, Feng Q, Qian Q, Li JY, Han B (2012). A map of rice genome variation reveals the origin of cultivated rice. Nature.

[32] Zhao KY, Tung CW, Eizenga GC, Wright MH, Liakat Ali M, Price AH, Norton GJ, Rafiqul Islam M, Reynolds A, Mezey J, McClung AM, Bustamante CD, McCouch SR (2011). Genome-wide association mapping reveals a rich genetic architecture of complex traits in *Oryza sativa*. Nat Commun.

[33] Chen W, Gao YQ, Xie WB, Gong L, Lu K, Wang WS, Li Y, Liu XQ, Zhang HY, Dong HX, Zhang W, Zhang LJ, Yu SB, Wang GW, Lian XM, Luo J (2014). Genome-wide association analyses provide genetic and biochemical insights into natural variation in rice metabolism. Nat Genet.

[34] Chen HD, Xie WB, He H, Yu HH, Chen W, Li J, Yu RB, Yao Y, Zhang WH, He YQ, Tang XY, Zhou FS, Deng XW, Zhang QF (2014). A high-density SNP genotyping array for rice biology and molecular breeding. Mol Plant.

[35] Yu HH, Xie WB, Li J, Zhou FS, Zhang QF (2014). A whole-genome SNP array (RICE6K) for genomic breeding in rice. Plant Biotechnol J.

[36] Nakagawa S (2004). A farewell to Bonferroni: the problems of low statistical power and publication bias. Behav Ecol.

[37] Gingerich DK, Gagne JM, Salter DW, Hellmann H, Estelle M, Ma LG, Vierstra RD (2005). Cullins 3a and 3b assemble with members of the Broad Complex/Tramtrack/Bric-a-Brac (BTB) protein family to form essential ubiquitin-protein ligases (E3s) in Arabidopsis. J Biol Chem.

[38] Hellmann H, Estellen M (2002). Plant development: regulation by protein degradation. Science.

[39] van der Knaap E, Kim JH, Kende H (2000). A novel gibberellin-induced gene from rice and its potential regulatory role in stem growth. Plant Physiol.

[40] Choi D, Kim JH, Kende H (2004). Whole genome analysis of the *OsGRF* gene family encoding plant-specific putative transcription activators in rice (*Oryza sativa* L.). Plant Cell Physiol.

[41] Berry PM, Sterling M, Spink JH, Baker CJ, Sylvester-Bradley R, Mooney SJ, Tams AR, Ennos AR (2004). Understanding and reducing lodging in cereals. Adv Agron.

[42] Cai HW, Morishima H (2002). QTL clusters reflect character associations in wild and cultivated rice. Theor Appl Genet.

[43] Benjamini Y, Hochberg Y (1995). Controlling the false discovery rate: a practical and powerful approach to multiple testing source. J R Stat Soc Ser B.

[44] Zhang HL, Zhang DL, Wang MX, Sun JL, Qi YW, Li JJ, Wei XH, Han LZ, Qiu AG, Tang SX, LZC (2011). A core collection and mini core collection of *Oryza sativa* L. in China. Theor Appl Genet.

[45] Luo LJ (2010). Breeding for water-saving and drought-resistance rice (WDR) in China. J Exp Bot.

[46] Li H, Durbin R (2009). Fast and accurate short read alignment with Burrows-Wheeler transform. Bioinformatics.

[47] Li H, Handsaker B, Wysoker A, Fennell T, Ruan J, Homer N, Marth G, Abecasis G, Durbin R, 1000 Genome Project Data Processing Subgroup (2009). The sequence alignment/map format and SAMtools. Bioinformatics.

[48] Yuan QP, Ouyang S, Wang AH, Zhu W, Maiti R, Lin HN, Hamilton J, Haas B, Sultana R, Cheung F, Wortman J, Buell R (2005). The Institute for Genomic Research Osa1 rice genome annotation database. Plant Physiol.

[49] Ouyang S, Zhu W, Hamilton J, Lin HN, Cambell M, Childs K, Thibaud-Nissen G, Malek RL, Lee YD, Zheng L, Orvis J, Haas B, Wortman J, Buell CR (2007). The TIGR rice genome annotation resource: improvements and new features. Nucleic Acids Res.

[50] Felsenstein J (1989). PHYLIP -Phylogeny inference package (version 3.2). Cladistics.

[51] Letunic I, Bork P (2011). Interactive Tree of Life v2: online annotation and display of phylogenetic trees made easy. Nucleic Acids Res.

[52] Pritchard JK, Stephens M, Donnelly P (2000). Inference of population structure using multilocus genotype data. Genetics.

[53] Falush D, Stephens M, Pritchard JK (2003). Inference of population structure using multilocus genotype data: linked loci and correlated allele frequencies. Genetics.

[54] Evanno G, Regnaut S, Goudet J (2005). Detecting the number of clusters of individuals using the software STRUCTURE: a simulation study. Mol Ecol.

[55] Zhang ZW, Ersoz E, Lai CQ, Todhunter RJ, Tiwari HK, Gore MA, Bradbury PJ, Yu JM, Arnett DK, Ordova JM, Buckler ES (2010). Mixed linear model adapted for genome-wide association studies. Nat Genet.

[56] Lipka AE, Tian F, Wang QS, Peiffer J, Li M, Bradbury PJ, Gore MA, Buckler ES, Zhang ZW (2012). GAPIT: genome association and prediction integrated tool. Bioinformatics.

