# Supplementary material for: Genome-wide Association Study (GWAS) of mesocotyl elongation based on re-sequencing approach in rice
Source: BMC Plant Biol. 2015 Sep 11;15:218. doi: 10.1186/s12870-015-0608-0 (PMC4566844; doi:10.1186/s12870-015-0608-0)
Supplement: Additional file 1: Table S1. — Pearson correlation coefficients between mesocotyl elongation and agronomic traits of mature plants measured in phenotyping trial with water regimes. (DOCX 16 kb) [file 12870_2015_608_MOESM1_ESM.docx]

Table S1 Pearson correlation coefficients between mesocotyl elongation and agronomic traits of mature plants measured in phenotyping trial with water regimes

| Traits | Treatments | MLw | MLs |
| --- | --- | --- | --- |
| MLs |  | 0.784** |  |
| Plant height | Water | 0.299** | 0.349** |
|  | Drought | 0.250** | 0.318** |
| Grain yield | Water | -0.007 | -0.079 |
|  | Drought | 0.239** | 0.188** |
|  |  |  |  |
| Spikelet fertility | Water | 0.010 | 0.099 |
|  | Drought | 0.227** | 0.188** |
|  |  |  |  |

MLw and MLs: mesocotyl length measured in dark germination and sand culture, respectively. Water and drought: well watered treatment and drought stress treatment. * and ** represent the significant level at 0.05, 0.01, respectively.
