# Supplementary material for: Genome-wide Association Study (GWAS) of mesocotyl elongation based on re-sequencing approach in rice
Source: BMC Plant Biol. 2015 Sep 11;15:218. doi: 10.1186/s12870-015-0608-0 (PMC4566844; doi:10.1186/s12870-015-0608-0)
Supplement: Additional file 2: Table S2. — Accuracy of SNP calling and missing genotype imputation validated by RiceSNP50 whole-genome SNP array. (DOCX 16 kb) [file 12870_2015_608_MOESM2_ESM.docx]

Table S2 Accuracy of SNP calling and missing genotype imputation validated by RiceSNP50 array

| Acc No | Name | Missing SNPs | Common SNPs | Consistent SNPs | Accuracy (%) |
| --- | --- | --- | --- | --- | --- |
| S11E0343 | TAINUNG 67 | 105 | 10746 | 10695 | 99.53 |
| S11E0348 | CYPRESS | 355 | 10496 | 10436 | 99.43 |
| S11E0349 | M 202 | 344 | 10507 | 10440 | 99.36 |
| S11E0201 | DULAR | 748 | 10103 | 10026 | 99.24 |
| S11E0175 | N 22 | 768 | 10083 | 10005 | 99.23 |
| S11E0265 | AIJIAONANTE | 570 | 10281 | 10198 | 99.19 |
| S11E0205 | BENBANGGU | 549 | 10302 | 10216 | 99.17 |
| S11E0026 | XIAOHONGGU | 589 | 10262 | 10171 | 99.11 |
| S11E0167 | XIBAINIAN | 540 | 10311 | 10189 | 98.82 |
| S11E0307 | ZHONG 413 | 742 | 10109 | 9982 | 98.74 |
| S11E0227 | LUHAN 1 | 801 | 10050 | 9904 | 98.55 |
| S11E0076 | MOWANGGU | 692 | 10159 | 9992 | 98.36 |
| S11E0301 | IRAT 109 | 607 | 10244 | 10071 | 98.31 |
| S11E0059 | BICO PRETO | 808 | 10043 | 9869 | 98.27 |
| S11E0043 | IAC 1246 | 657 | 10194 | 10016 | 98.25 |
| S11E0113 | CICA 4 | 649 | 10202 | 10019 | 98.21 |
| S11E0225 | LAC 23 | 618 | 10233 | 10047 | 98.18 |
| S11E0292 | ZHENSHAN 97B | 499 | 10352 | 10152 | 98.07 |
| S11E0075 | ZHONGHAN 3 | 833 | 10018 | 9788 | 97.70 |
| S11E0103 | IAC 1 | 867 | 9984 | 9752 | 97.68 |
| S11E0192 | TRES MESES | 1172 | 9679 | 9408 | 97.20 |
| S11E0109 | QINGSIZHAN 1 | 1825 | 9026 | 8769 | 97.15 |
| S11E0093 | PR 325 | 2538 | 8313 | 8065 | 97.02 |
| S11E0135 | IR 30358-084-1-1 | 1180 | 9671 | 9382 | 97.01 |
| Means |  | 794 | 10057 | 9899.67 | 98.41 |
