# Supplementary figures and images for: Genome-wide Association Study (GWAS) of mesocotyl elongation based on re-sequencing approach in rice
Source: BMC Plant Biol. 2015 Sep 11;15:218. doi: 10.1186/s12870-015-0608-0 (PMC4566844; doi:10.1186/s12870-015-0608-0)

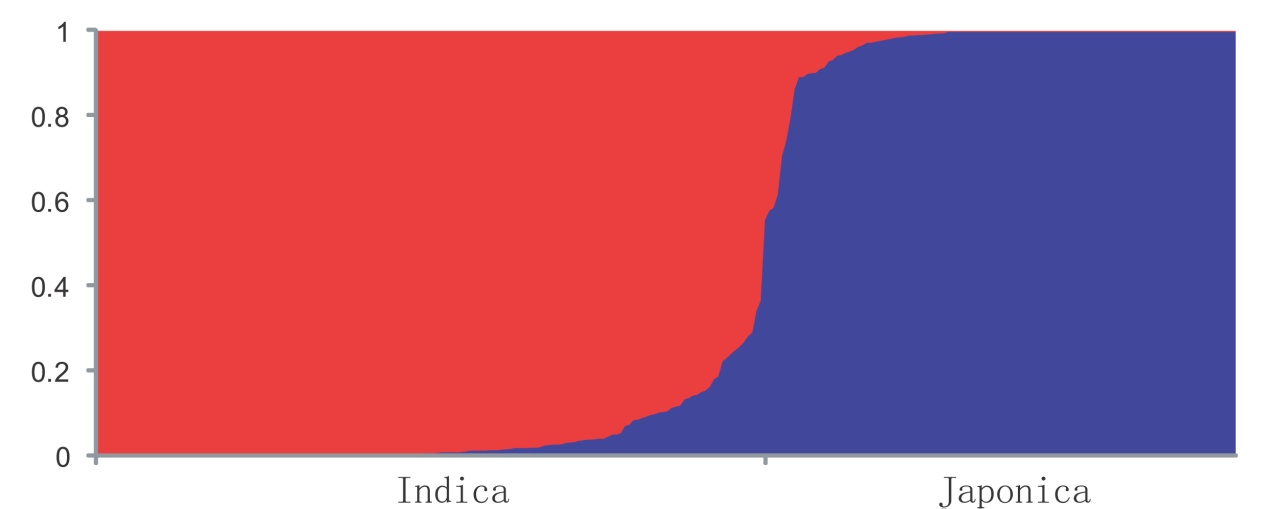


Figure S1 Two subpopulations defined by STRUCTURE

Supplement: Additional file 3: Figure S1. — Two subpopolations defined by STRUCTURE. (DOCX 51 kb) [file 12870_2015_608_MOESM3_ESM.docx]
