# Supplementary material for: Genome-wide Association Study (GWAS) of mesocotyl elongation based on re-sequencing approach in rice
Source: BMC Plant Biol. 2015 Sep 11;15:218. doi: 10.1186/s12870-015-0608-0 (PMC4566844; doi:10.1186/s12870-015-0608-0)
Supplement: Additional file 4: Table S3. — Model selection results for GWAS of rice mesocotyl lengths in two experiments. (DOCX 15 kb) [file 12870_2015_608_MOESM4_ESM.docx]

Table S3 Model selection results for GWAS of rice mesocotyl lengths in two experiments

| Number of PCs/Covariates | BIC (larger is better) - Schwarz 1978 | |
| --- | --- | --- |
|  | MLw | MLs |
| 0 | **-732.55** | **-603.79** |
| 1 | -735.09 | -606.2 |
| 2 | -737.7 | -608.85 |
| 3 | -739.6 | -609.22 |
| 4 | -741.9 | -610.81 |
| 5 | -744.61 | -613.44 |
| 6 | -747.31 | -615.74 |
| 7 | -750.03 | -618.24 |
| 8 | -743.98 | -613.71 |
| 9 | -741.63 | -613.39 |
| 10 | -743.94 | -615.83 |

MLw and MLs: mesocotyl lengths (cm) measured in dark germination with water or in 5cm sand culture, respectively.
