# Supplementary material for: Genome-wide Association Study (GWAS) of mesocotyl elongation based on re-sequencing approach in rice
Source: BMC Plant Biol. 2015 Sep 11;15:218. doi: 10.1186/s12870-015-0608-0 (PMC4566844; doi:10.1186/s12870-015-0608-0)
Supplement: Additional file 6: Figure S2. — Distribution of –log(P) and –log(FDR adjusted P) values of SNPs with –log(FDR adjusted P) ≥3.0 (A) and the parallel changes of both parameters estimated in GWAS of MLw. (DOCX 126 kb) [file 12870_2015_608_MOESM6_ESM.docx]

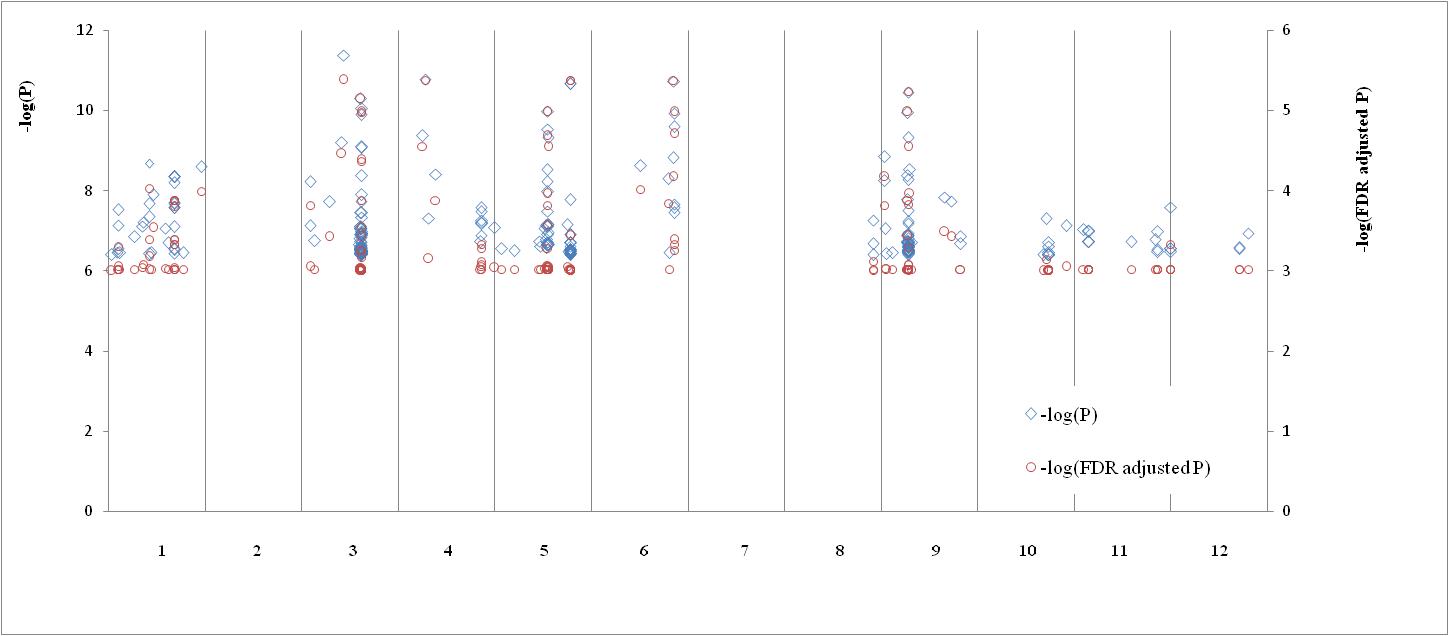


Chromosome

A


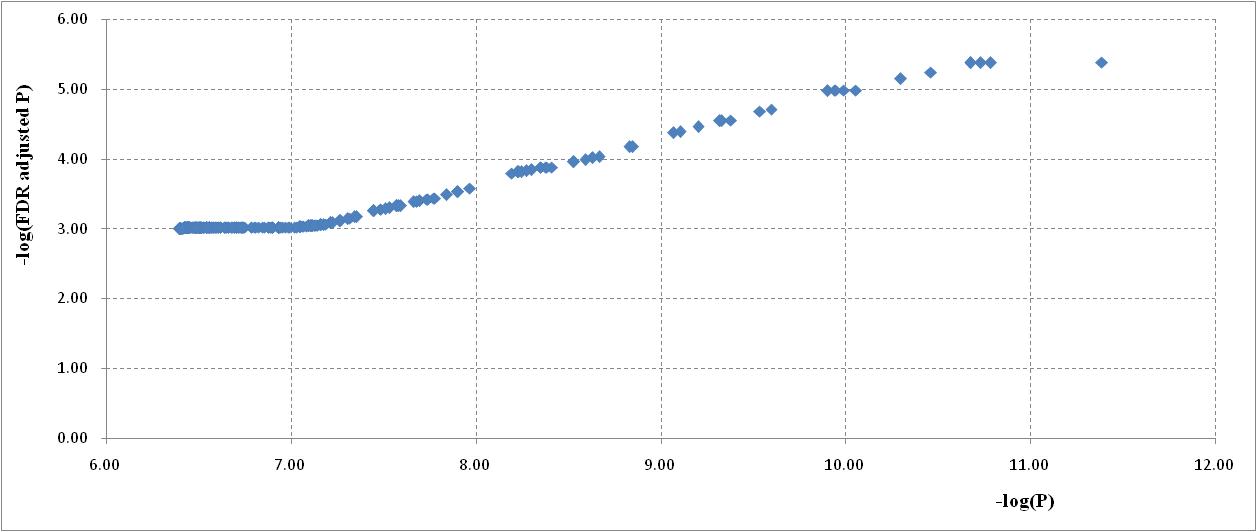


B

## Figure S2. Distribution of –log(P) and –log(FDR adjusted P) values of SNPs with –log(FDR adjusted P)≥3.0 (A) and the parallel changes of both parameters estimated in GWAS of MLw

The positions of SNPs were presented in relative values against the lengths of each chromosome. So the abscissa axis did not show the real lengths of chromosomes.
