# Supplementary material for: Genome-wide Association Study (GWAS) of mesocotyl elongation based on re-sequencing approach in rice
Source: BMC Plant Biol. 2015 Sep 11;15:218. doi: 10.1186/s12870-015-0608-0 (PMC4566844; doi:10.1186/s12870-015-0608-0)
Supplement: Additional file 7: Table S5. — List of rice landraces or varieties used in this study. (DOCX 33 kb) [file 12870_2015_608_MOESM7_ESM.docx]

Table S5 List of rice landraces or varieties used in this study

| Acc. No. | C/D collection | Name | *Indica*/*japonica*/*aus* |
| --- | --- | --- | --- |
| S11E0001 | C | XIMAXIAN | *japonica* |
| S11E0002 | C | YUYANNUO | *japonica* |
| S11E0003 | C | CHENWAN 3 | *indica* |
| S11E0004 | C | HONGWAN 1 | *indica* |
| S11E0005 | D | HAOHAI | *japonica* |
| S11E0006 | C | TIEGANWU | *japonica* |
| S11E0007 | C | HONGAINUO | *indica* |
| S11E0009 | C | NANGAOGU | *indica* |
| S11E0010 | C | HONGMISANDAN | *japonica* |
| S11E0012 | C | WUZUIHONGGU | *indica* |
| S11E0013 | C | BEIZINUO | *indica* |
| S11E0014 | D | HUHAN 2B | *japonica* |
| S11E0015 | D | ZAOHANDAO | *japonica* |
| S11E0017 | D | C 22 | *indica* |
| S11E0018 | D | HAOGANG | *japonica* |
| S11E0019 | C | MILYANG 23 | *indica* |
| S11E0020 | C | PEI C122 | *japonica* |
| S11E0021 | C | MOMI | *indica* |
| S11E0022 | C | YANGDAO 2 | *indica* |
| S11E0023 | C | GENG 87-304 | *japonica* |
| S11E0024 | C | SUGENG 2 | *japonica* |
| S11E0025 | C | LAOHUZHONG | *japonica* |
| S11E0026 | C | XIAOHONGGU | *indica* |
| S11E0027 | C | XIANGGU | *indica* |
| S11E0028 | C | WUZIDUI | *japonica* |
| S11E0029 | C | BIWUSHENG | *indica* |
| S11E0030 | C | YIZHIXIANG | *indica* |
| S11E0031 | D | SILEWAH | *japonica* |
| S11E0032 | D | DINALAGA | *indica* |
| S11E0033 | D | IAC 5100 | *indica* |
| S11E0034 | D | MILT 1444 | *japonica* |
| S11E0035 | D | T 1095 | *japonica* |
| S11E0037 | D | ZHAXIMA | *japonica* |
| S11E0038 | D | BAYUENUO | *japonica* |
| S11E0039 | C | HONGKEZHENUO | *japonica* |
| S11E0040 | D | IRAT 13 | *japonica* |
| S11E0041 | D | KN 361-1-8-6 | *japonica* |
| S11E0042 | D | IR 75942-9 | *indica* |
| S11E0043 | D | IAC 1246 | *japonica* |
| S11E0044 | D | TORIDE 1 | *japonica* |
| S11E0047 | D | KU 70-1 | *japonica* |
| S11E0048 | D | MIFOR 6-2 | *japonica* |
| S11E0049 | C | HAOBUKA | *japonica* |
| S11E0050 | C | LIXINGENG | *japonica* |
| S11E0051 | C | JINZHINUO | *indica* |
| S11E0052 | C | QITOUGU | *indica* |
| S11E0053 | C | MINBEIWANXIAN | *indica* |
| S11E0054 | D | LAMBAYEQUE 1 | *aus* |
| S11E0055 | D | UVS | *japonica* |
| S11E0056 | D | WANHANDAO | *japonica* |
| S11E0057 | C | JWR 221 | *indica* |
| S11E0058 | C | YANGKENUO | *japonica* |
| S11E0059 | D | BICO PRETO | *japonica* |
| S11E0061 | C | AZUCENA | *japonica* |
| S11E0062 | C | XIUSHUI 115 | *japonica* |
| S11E0063 | C | DANGYU 5 | *indica* |
| S11E0065 | C | ZHENXIAN 232 | *indica* |
| S11E0067 | D | NAVOLATO A 71 | *japonica* |
| S11E0068 | D | DNJ 171 | *japonica* |
| S11E0071 | D | JAPPENI TUNGKUNGO | *indica* |
| S11E0072 | D | AUS 454 | *aus* |
| S11E0074 | D | IGUAPE CATETO | *japonica* |
| S11E0075 | D | ZHONGHAN 3 | *japonica* |
| S11E0076 | D | MOWANGGU | *japonica* |
| S11E0077 | C | CHENGNONGSHUIJING | *indica* |
| S11E0079 | C | XIANGNUO | *japonica* |
| S11E0080 | C | WH139 | *indica* |
| S11E0081 | C | MINGHUI 63 | *indica* |
| S11E0082 | C | AIMI | *indica* |
| S11E0083 | D | KHAU MEO | *indica* |
| S11E0084 | D | HANDAO 3 | *japonica* |
| S11E0086 | D | MONOLAYA | *indica* |
| S11E0088 | D | BPI 9-33 | *indica* |
| S11E0093 | D | PR 325 | *indica* |
| S11E0095 | D | GANLANGU | *japonica* |
| S11E0097 | D | COLOMBIA 1 | *indica* |
| S11E0098 | D | NORIN 24 | *indica* |
| S11E0099 | D | JINHUANGZHAN | *indica* |
| S11E0100 | D | EMATA YIN | *japonica* |
| S11E0101 | D | DT001 | *indica* |
| S11E0102 | D | DOURADO AGULHA | *indica* |
| S11E0103 | D | IAC 1 | *japonica* |
| S11E0104 | D | IR 442-2-58 | *japonica* |
| S11E0105 | D | II-32 B | *indica* |
| S11E0106 | C | NINGHUI 21 | *japonica* |
| S11E0107 | D | BENGUE | *japonica* |
| S11E0109 | D | QINGSIZHAN 1 | *indica* |
| S11E0111 | D | DJAUB | *japonica* |
| S11E0112 | D | DALAI AMAN | *indica* |
| S11E0113 | D | CICA 4 | *japonica* |
| S11E0114 | D | ZHENFU | *indica* |
| S11E0116 | D | MIGA | *japonica* |
| S11E0117 | D | IR 10781-75-3-2-2 | *japonica* |
| S11E0118 | D | BALA | *indica* |
| S11E0119 | D | MRC 172-9 | *indica* |
| S11E0120 | D | LIUHUANGZHAN | *indica* |
| S11E0122 | C | GUIHUAHUANG | *japonica* |
| S11E0124 | C | GU 154 | *indica* |
| S11E0126 | C | LENGSHUIGU 2 | *japonica* |
| S11E0127 | C | 02428 | *japonica* |
| S11E0128 | C | TAICHUNG NATIVE 1 | *indica* |
| S11E0131 | D | DS001 | *indica* |
| S11E0132 | D | SML 81B | *indica* |
| S11E0133 | D | ITA 141 | *japonica* |
| S11E0135 | D | IR 30358-084-1-1 | *indica* |
| S11E0136 | D | DAYEZAO | *indica* |
| S11E0137 | D | JUMALI | *japonica* |
| S11E0140 | C | GUICHAO 2 | *indica* |
| S11E0142 | C | BAWANGBIAN 1 | *indica* |
| S11E0143 | C | WH62 | *indica* |
| S11E0144 | C | TAIZHONGXIANXUAN 2 | *indica* |
| S11E0145 | C | AITUOGU 151 | *indica* |
| S11E0146 | C | 76-1 | *indica* |
| S11E0147 | D | BICO BRANCO | *indica* |
| S11E0148 | D | LIANGGUOZAO | *indica* |
| S11E0149 | D | DUOCHE | *indica* |
| S11E0150 | D | IPEACO 162 | *japonica* |
| S11E0153 | D | YUNLU 8 | *japonica* |
| S11E0154 | D | PR 403 | *indica* |
| S11E0156 | C | MOROBEREKAN | *indica* |
| S11E0157 | C | 88B | *indica* |
| S11E0158 | C | SANKECUN | *indica* |
| S11E0159 | C | TEQINGXUANHUI | *indica* |
| S11E0160 | C | JINYOU 1 | *indica* |
| S11E0161 | C | DONGTINGWANXIAN | *indica* |
| S11E0162 | C | HANMADAO | *indica* |
| S11E0163 | C | SHANJIUGU | *japonica* |
| S11E0164 | C | SANLICUN | *indica* |
| S11E0165 | C | XIANGAI B | *indica* |
| S11E0166 | C | MENJIADING 2 | *indica* |
| S11E0167 | C | XIBAINIAN | *indica* |
| S11E0168 | C | CUNGUNUO | *japonica* |
| S11E0171 | D | KEQING 3 | *indica* |
| S11E0172 | D | RIKUTO NORIN 21 | *indica* |
| S11E0173 | D | HEGANXIANNIAN | *indica* |
| S11E0174 | C | SHANHUANGZHAN 2 | *indica* |
| S11E0175 | C | N 22 | *aus* |
| S11E0176 | C | QINGSIAI 16B | *indica* |
| S11E0177 | C | JIABALA | *indica* |
| S11E0178 | C | TAISHANNUO | *indica* |
| S11E0179 | C | HUANGSIGUIZHAN | *indica* |
| S11E0180 | C | XIANGWANXIAN 3 | *indica* |
| S11E0181 | C | ESINIU | *indica* |
| S11E0182 | C | CUNSANLI | *japonica* |
| S11E0183 | C | GAOYANGDIANDAO | *japonica* |
| S11E0184 | C | CHIKENUO | *japonica* |
| S11E0185 | C | XUGUNUO | *indica* |
| S11E0186 | C | AIMAKANG | *indica* |
| S11E0187 | C | BAIKEZAOHE | *indica* |
| S11E0188 | C | XIAOBAIMI | *indica* |
| S11E0190 | D | BLUE BELLE | *japonica* |
| S11E0191 | D | IR 65907-116-1-B | *indica* |
| S11E0192 | D | TRES MESES | *indica* |
| S11E0193 | D | TAITUNG 16 | *indica* |
| S11E0194 | D | NEP HUONG | *indica* |
| S11E0195 | D | WUJIANNEITIANGU | *indica* |
| S11E0196 | D | TODOROKIWASE | *japonica* |
| S11E0197 | D | CARTUNA | *indica* |
| S11E0198 | D | IR 1487-372-4 | *indica* |
| S11E0199 | D | BINIRHEN | *japonica* |
| S11E0200 | D | IR 66417-18-1-1-1 | *indica* |
| S11E0201 | C | DULAR | *aus* |
| S11E0202 | C | DIANRUI 409B | *indica* |
| S11E0203 | C | ZAOSHUNONGHU 6 | *japonica* |
| S11E0204 | C | LIUSHA 1 | *indica* |
| S11E0205 | C | BENBANGGU | *indica* |
| S11E0206 | C | QITOUBAIGU | *indica* |
| S11E0207 | C | MEIHUANUO | *indica* |
| S11E0209 | C | WH109 | *indica* |
| S11E0210 | C | JIANGNONGZAO 1 | *indica* |
| S11E0211 | C | 93-11 | *indica* |
| S11E0212 | C | ZHUZHEN B | *indica* |
| S11E0213 | C | ZAOSHUXIANGHEI | *indica* |
| S11E0214 | C | NANJING 11 | *indica* |
| S11E0215 | C | GUI 630 | *indica* |
| S11E0216 | C | XIANGWANXIAN 1 | *indica* |
| S11E0217 | C | XINGGUO | *japonica* |
| S11E0218 | C | HANGXIANLIANGCHUN | *indica* |
| S11E0219 | C | MAWEINIAN | *indica* |
| S11E0220 | D | HANDAO 8 | *japonica* |
| S11E0225 | D | LAC 23 | *japonica* |
| S11E0226 | D | PRATAO | *japonica* |
| S11E0227 | D | LUHAN 1 | *indica* |
| S11E0228 | C | MUGUANUO | *japonica* |
| S11E0229 | C | HEIDU 4 | *indica* |
| S11E0230 | C | SHUFENG 101 | *indica* |
| S11E0231 | C | CHENGDUAI 3 | *indica* |
| S11E0232 | C | GONGJU 73 | *indica* |
| S11E0233 | C | DANDONGLUDAO | *japonica* |
| S11E0234 | C | MUXIQIU | *japonica* |
| S11E0235 | C | NANTEHAO | *indica* |
| S11E0236 | C | HUANGKEZAONIAN | *japonica* |
| S11E0237 | C | LIMING B | *japonica* |
| S11E0238 | C | JIEFANGXIAN | *indica* |
| S11E0239 | C | WANLIXIAN | *indica* |
| S11E0240 | C | ZHONGHUA 11 | *japonica* |
| S11E0241 | D | ZHONGHAN 209 | *indica* |
| S11E0242 | C | LEIHUOZHAN | *indica* |
| S11E0243 | C | PUTAOHUANG | *japonica* |
| S11E0245 | C | GERDEH | *japonica* |
| S11E0246 | C | LIJIANGXINTUANHEIGU | *japonica* |
| S11E0247 | C | WEIGUO | *japonica* |
| S11E0248 | C | ZHONGLOU 1 | *japonica* |
| S11E0250 | C | GZHENSHAN 97B | *indica* |
| S11E0251 | C | IR 661-1 | *indica* |
| S11E0253 | C | TAIDONGLUDAO | *japonica* |
| S11E0254 | C | BALILLA | *japonica* |
| S11E0255 | C | LIAOGENG 287 | *japonica* |
| S11E0256 | C | ZHONGHUA 8 | *japonica* |
| S11E0257 | C | SADU CHO | *indica* |
| S11E0259 | C | BAOXIE 7B | *indica* |
| S11E0260 | C | MAGUZI | *japonica* |
| S11E0261 | C | JINDAO 1 | *japonica* |
| S11E0262 | C | YOUMANGZAOGENG | *japonica* |
| S11E0263 | C | SHUIYUAN300LI | *japonica* |
| S11E0264 | C | GUANGLUAI 15 | *indica* |
| S11E0265 | C | AIJIAONANTE | *indica* |
| S11E0266 | C | GUANGLUAI4 | *indica* |
| S11E0267 | C | XIANGAIZAO 10 | *indica* |
| S11E0268 | C | FUNINGZIPI | *japonica* |
| S11E0269 | C | WH115 | *indica* |
| S11E0270 | C | YELICANGHUA | *japonica* |
| S11E0271 | C | CHAOYANG 1B | *indica* |
| S11E0274 | C | LONGHUAMAOHU | *japonica* |
| S11E0275 | C | ERJIUNAN 1 | *indica* |
| S11E0276 | C | L 301B | *indica* |
| S11E0277 | C | JINNANTEB | *indica* |
| S11E0278 | C | ANNONGWANGENG B | *japonica* |
| S11E0279 | C | SANBAILI | *indica* |
| S11E0280 | C | LAOGUANGTOU 83 | *japonica* |
| S11E0281 | C | HEIGENG 2 | *japonica* |
| S11E0282 | C | NANXIONGZAOYOU | *indica* |
| S11E0283 | D | C 418 | *japonica* |
| S11E0284 | D | HUHAN 1B | *indica* |
| S11E0285 | D | HUHAN 7B | *indica* |
| S11E0286 | D | HANHUI 3 | *indica* |
| S11E0287 | D | XIANGQING | *japonica* |
| S11E0288 | D | HUHAN 3 | *japonica* |
| S11E0289 | D | HUHAN 15 | *indica* |
| S11E0290 | D | XIUSHUI 123 | *japonica* |
| S11E0291 | D | HANHUI 15 | *indica* |
| S11E0292 | D | ZHENSHAN 97B | *indica* |
| S11E0300 | D | TEQING | *indica* |
| S11E0301 | D | IRAT 109 | *japonica* |
| S11E0307 | D | ZHONG 413 | *indica* |
| S11E0314 | C | SANBANGQISHILUO | *japonica* |
| S11E0315 | C | AIHECHI | *indica* |
| S11E0316 | C | HUANGPINUO | *japonica* |
| S11E0318 | C | JINXIBAI | *indica* |
| S11E0319 | C | BABAILI | *japonica* |
| S11E0320 | C | HAOMAKE(K) | *japonica* |
| S11E0321 | C | HAOBAYONG 1 | *japonica* |
| S11E0323 | C | NIANKENUO | *japonica* |
| S11E0326 | C | YOUNIAN | *indica* |
| S11E0328 | C | MAMAGU | *indica* |
| S11E0329 | C | XIANGDAO | *indica* |
| S11E0330 | C | FEIDONGTANGDAO | *japonica* |
| S11E0331 | C | ZEGU | *indica* |
| S11E0333 | C | BAIKEHUALUO | *indica* |
| S11E0334 | C | ZHONGNONG 4 | *indica* |
| S11E0335 | C | LUCAIHAO | *indica* |
| S11E0336 | C | ZAOXIAN 240 | *indica* |
| S11E0337 | C | JINNANTE 43B | *indica* |
| S11E0338 | C | XIANGZAOXIAN 7 | *indica* |
| S11E0340 | C | 80B | *indica* |
| S11E0342 | C | MOWANGGUNEI | *indica* |
| S11E0343 | C | TAINUNG 67 | *japonica* |
| S11E0345 | C | NIPPONBARE | *japonica* |
| S11E0348 | C | CYPRESS | *japonica* |
| S11E0349 | C | M 202 | *japonica* |
